# Classification of endogenous and exogenous bursts in collective emotions based on Weibo comments during COVID-19

**DOI:** 10.1038/s41598-022-07067-w

**Published:** 2022-02-24

**Authors:** Qianyun Wu, Yukie Sano, Hideki Takayasu, Misako Takayasu

**Affiliations:** 1 Department of Mathematical and Computing Science, School of Computing, Tokyo Institute of Technology, Yokohama, 226‑8502 Japan; 2 Faculty of Engineering, Information and Systems, University of Tsukuba, Tsukuba, 305-8573 Japan; 3 Sony Computer Science Laboratories, Tokyo, 141‑0022 Japan; 4 Institute of Innovative Research, Tokyo Institute of Technology, Yokohama, 226‑8502 Japan

**Keywords:** Applied mathematics, Statistics, Statistical physics, thermodynamics and nonlinear dynamics

## Abstract

Bursts and collective emotion have been widely studied in social physics field where researchers use mathematical models to understand human social dynamics. However, few researches recognize and separately analyze the internal and external influence on burst behaviors. To bridge this gap, we introduce a non-parametric approach to classify an interevent time series into five scenarios: random arrival, endogenous burst, endogenous non-burst, exogenous burst and exogenous non-burst. In order to process large-scale social media data, we first segment the interevent time series into sections by detecting change points. Then we use the rule-based algorithm to classify the time series based on its distribution. To validate our model, we analyze 27.2 million COVID-19 related comments collected from Chinese social media between January to October 2020. We adopt the emotion category called Profile of Mood States which consists of six emotions: *Anger*, *Depression*, *Fatigue*, *Vigor*, *Tension* and *Confusion*. This enables us to compare the burst features of different collective emotions during the COVID-19 period. The burst detection and classification approach introduced in this paper can also be applied to analyzing other complex systems, including but not limited to social media, financial market and signal processing.

## Introduction

Heavy tailed phenomena have been observed in various human activities such as phone calls, emails, and social media communications^1–6^ including user’s comment posting behaviors^7,8^. We can observe similar comment posting behavior based on data obtained from Weibo, the leading social platform in China. When each user publishes a comment randomly and independently, comment’s arrival follows a Poisson process and the arrival duration follows an exponential distribution^3,6^. However, when the collective emotion emerges and the users behave dependently, the density of arrival may be clustered and the distribution function of interarrival times may show a heavy tail which is typically approximated by a power-law distribution^1–3,5–8^.

On one hand, we argue that a burst can be triggered by emotional empathy among users when they browse comments published in the social media platform. When a user is exposed to comments over a certain period, he or she shares the same feeling and leave a comment. This behavior is widely studied using different approaches, for example, statistical hypothesis testing for examining factors influencing emotion changes^9,10^, virus models which regard the spread of emotions as infectious disease^11–15^, agent-based models for simulating the interactions of individual users^4,16,17^, and network theories that study the flow of emotion and information in the social networks^1,18^. On the other hand, bursts are considered to be triggered by external sources such as news, government propaganda, or extraordinary events. For example, when users are exposed to breaking news, they search for related posts and leave comments to express their emotions and opinions^12,19,20^.

We define the emotion empathy as endogenous factors influenced by precedent comments and exogenous factors triggered by external sources such as news. Although a few researchers have also considered the endogenous and exogenous influence on collective human behavior, most of them used parametric approaches that infer parameters for given models^12,20^. It is computationally expensive to run parametric analysis on long-term time series which is large in data size and whose distribution may change over time. In this paper we propose a non-parametric approach that can effectively detect endogenous and exogenous burst periods even from large dataset. More specifically, we first segment the time series to homogenous sections using the Fisher’s Exact Test^21^, which allows us to study the different comment arrival patterns separately. Then for each time series section, we presume that if the collective emotion is triggered by an endogenous factor, the corresponding time series should follow an autoregressive model that can be approximated by a non-stationary Poisson process called the self-modulation process^22^. Otherwise, we presume that the burst is caused by an exogenous factor.

Our paper introduces an empirical approach to detect and analyze collective emotion dynamics statistically focusing on each comment arrival. To validate its robustness, we deploy it to analyze 27.2 million COVID-19 related comments which were collected from the Weibo platform during January to October 2020. To our best knowledge, although there are numerous collective emotion researches related to COVID-19, most of them either visualize the emotion profiles in one or multiple countries^23,24^, or extract the most frequent topics and analyze their correlation to emotions^25,26^. Comparatively, however, fewer studies are done statistically to investigate the underlying dynamics which form the collective emotions. Yin et al.^27^ proposed an emotion-based susceptible-forwarding-immune model to study how is the public emotion shaped when users repost with comments. Velásquez et al.^28^ identified hate clusters in six social media platforms and used network theory to illustrate how malicious contents diffuses among different platforms.

Our approach to analyze collective emotion dynamics can be briefly summarized as follows. We build a multi-class classifier based on POMS (Profile of Mood States) and use it to identify emotion words from Weibo comments. The POMS has been traditionally used as a survey to rate a participant’s emotion states^29,30^, and has recently been used for analyzing public emotions in social media^31,32^. It consists of 6 emotion categories—*Anger*, *Depression*, *Fatigue*, *Vigor*, *Tension* and *Confusion*. POMS is considered to be suitable for analyzing the COVID-19 related emotions because people tend to react more negatively to the surroundings when exposed to the risk of infection^33^. Next, we introduce a non-parametric method to distinguish the endogenous and exogenous emotion bursts. Based on the burst analysis, we explain how do the popularity of emotions evolve and show the difference of burst features between different emotions.

## Results

**Figure 1:**
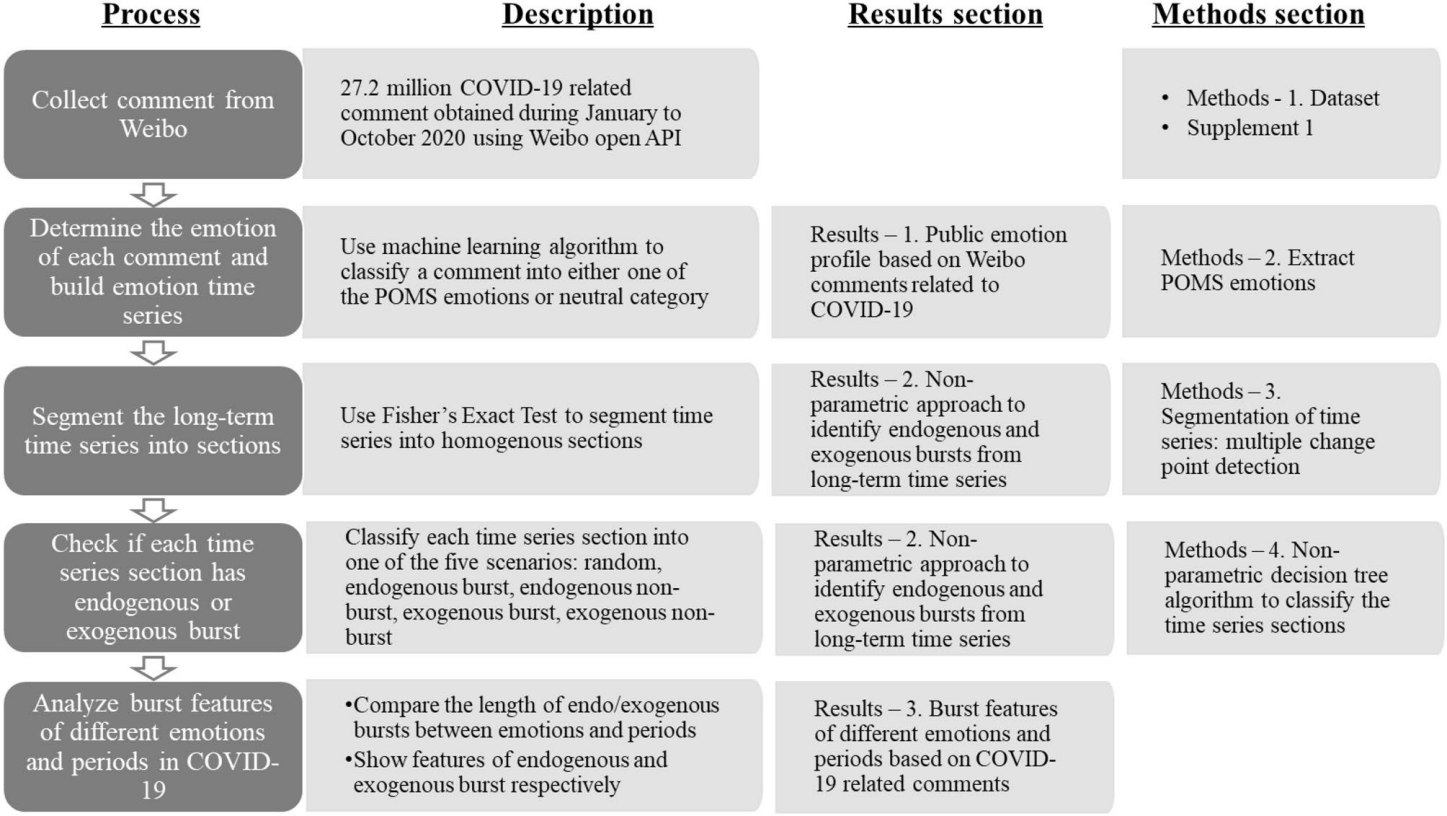
The overall data analysis process. It shows the steps to obtain data, analyze data and interpret analysis results. The results and details of methods can be found in respective sections as indicated in the figure.

In the Results section, we will explain the concept of a non-parametric approach to classify endogenous and exogenous bursts based on the emotion time series and show the result of empirical data analysis. Details of the methods will be provided in the “Methods” section. Figure 1 summarizes the data analysis process. It can also be used as a directory for reading this paper.

### Public emotion profile based on Weibo comments related to COVID-19

**Figure 2:**
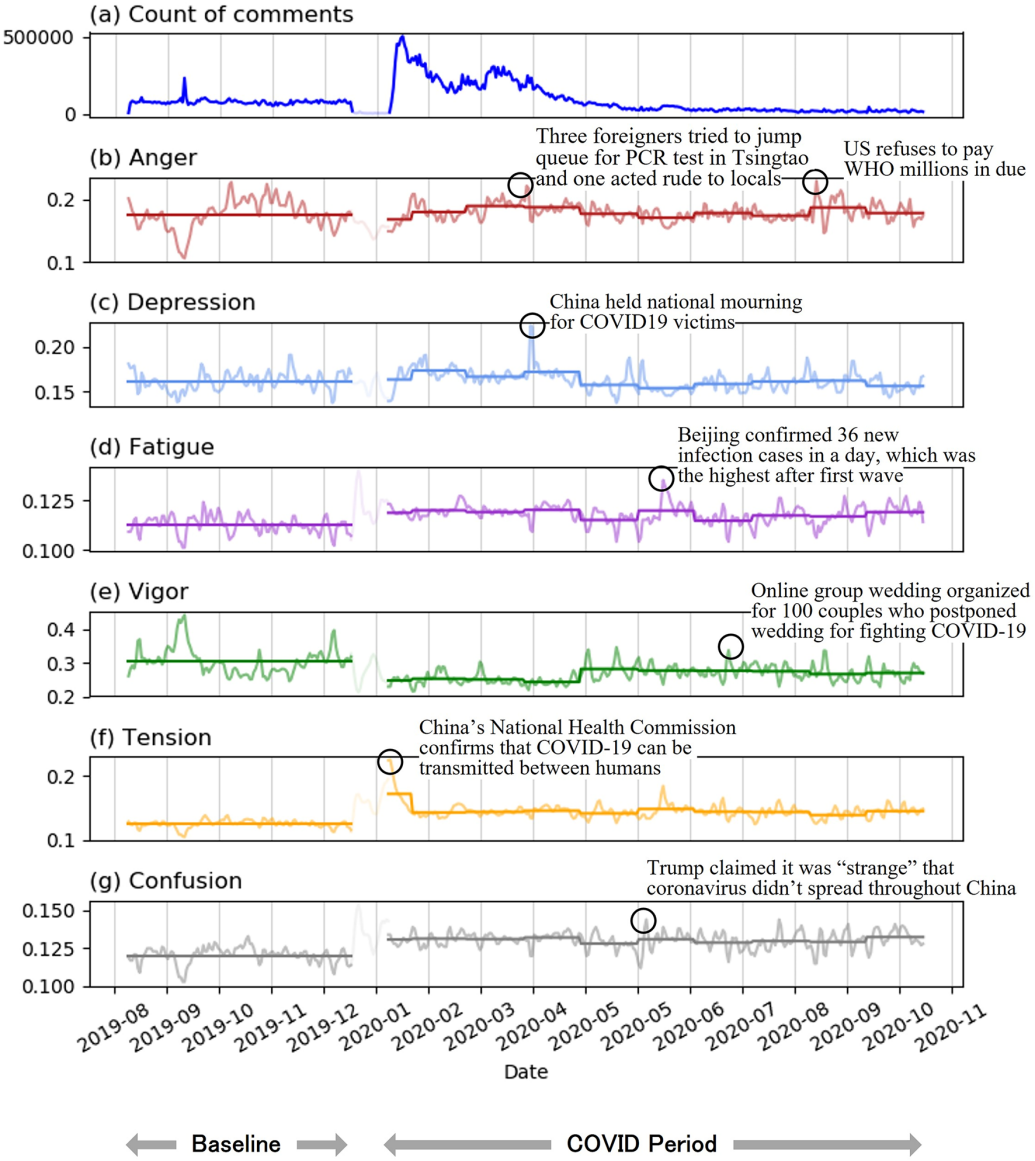
Normalized POMS emotion time series before and during the COVID-19 crisis in China. (**a**) The time series plot showing the daily number of comments related to COVID-19. (**b**–**g**) The time series plot showing the normalized daily number of comments under each emotion—*Anger, Depression, Fatigue, Vigor, Tension* and *Confusion*. The normalization is done by dividing the daily number of comments containing a specific emotion by the total daily number of comments. The key events that happened at the peak point of each time series plot are marked out.

We first visualize the public emotion profile (see Fig. 2) to give a general idea of how users in the Weibo platform responded to the COVID-19 crisis during January to October 2020.

We extract the POMS emotions from Weibo comments and analyze the daily count of comments under each emotion. Briefly, the emotion extraction method can be described as follows. We build a Chinese version of POMS dictionary containing 3944 emotional words categorized into 6 POMS emotions and 2500 neutral words (the corpus has been provided in Supplement 2). The dictionary is used as labeled data for training a multi-class emotion classifier that can automatically categorize a new word into *Anger, Depression, Fatigue, Vigor, Tension, Confusion* or *Neutral*. To extract emotions from Weibo comments, each comment is tokenized into words and then each word is processed using this classifier to determine its emotion category. After summarizing the frequency of emotion words for each comment, we take the emotion with the highest frequency as the emotion of the comment. The details of data and algorithms are provided in the “Methods” section.

Next, we build the time series for each emotion and normalize it by the daily total count of comments. We show the public emotion profile in three phases (see Fig. 2): the baseline period before the COVID-19 from August to December 2019, the initial discovery of the COVID-19 during early January 2020 and the outbreak from end January to October 2020. We excluded (shaded) the second phase because at that time the COVID-19 was neither officially reported nor widely discussed in social media which resulted in high fluctuation in normalized emotion time series.

When comparing emotions before and during the COVID-19, we can observe that *Anger* remained at the similar level and not much affected by the COVID-19 situation. *Depression* rose slightly in early outbreak period from January to April 2020 and fell back to the baseline level afterwards. *Fatigue* also rose during the COVID-19 compared to the baseline and remained at a higher level from January to October. *Vigor* decreased since the COVID-19 outbreak. Although it rose a bit after April 2020, the level was still lower than the baseline. *Tension* surged at the point when the COVID-19 was firstly confirmed by the government, but it immediately dropped despite the worsening COVID-19 situation. This may be explained by a phenomenon called psychological resilience^34^. When people are faced with a stressful situation, they tend to develop positive emotions with a moderate tension level. The overall *Tension* level during the COVID-19 was higher than the baseline. *Confusion* increased during the COVID-19 period compared to the baseline and it remained steady throughout the observation period.

### Non-parametric approach to identify endogenous and exogenous bursts from long-term time series

In this paper, we develop a non-parametric approach to reduce computation complexity. The method consists of two steps: (1) segment the time series into homogenous sections and (2) use hypothesis test to check if a time series section belongs to endogenous, exogenous burst or other arrival patterns.

Here in the Results section, we describe the concept and show the results of the non-parametric approach. The details are provided in the “Methods” section—4. Non-parametric rule-based algorithm to classify the time series sections.

1. Segment the long-term time series into homogenous sections

Because the user behavior may change over time which results in different distributions, we need to segment the long-term time series before doing the burst analysis. We introduce a new segmentation method applied to the comment count time series. We first aggregate the time series per 600 s to mitigate zero-valued data points while keeping the fluctuating pattern in the time series. However, the six emotion time series spanning over 10 months still contains more than 40,000 data points each, which is large in size. Therefore, a non-parametric segmentation method is preferred. We adopt a method based on the Fisher's Exact Test proposed by Sato and Takayasu^21^.

Conceptually, the Fisher’s Exact Test functions as follows. We start with the single point detection. If there exists one change point $\upsilon$ in a time series {${r}_{t}$}, then the time series segments before and after $\upsilon$ should be inhomogeneous, which can be tested using the Fisher’s Exact Test (the lower the p-value is, the less homogenous are the two datasets). Therefore, to look for a change point in a time series, we calculate the hypergeometric probability for all the points in a time series and take the minimum hypergeometric probability as the p-value. If this p-value is lower than the pre-determined threshold, then we adopt the corresponding time point $t$ as the change point $\upsilon$. Otherwise, we conclude that there is no change point in the given time series.

To extend this method to multiple change points detection, we simply need to repeat the same process recursively on the time series segments, until all segments’ p-values are higher than the threshold, which indicates that there exists no more change point.

**Figure 3:**
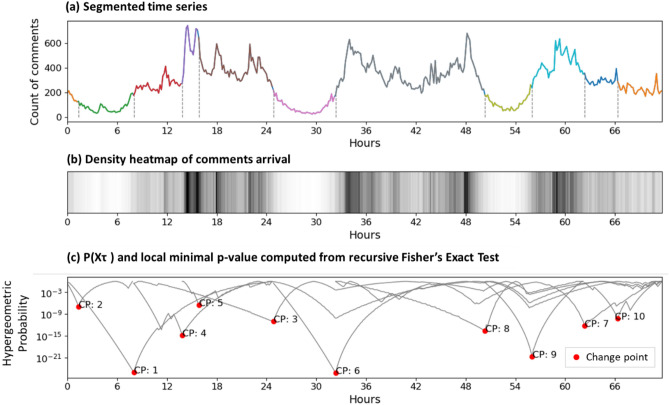
Time series segmentation using the Fisher’s Exact Test. (**a**) An example of segmentation result using proposed Fisher’s Exact Test. The x-axis is the timestamp (unit time 600 s) and the y-axis is the count of comments. Each segmented section is shown in different colors and separated using dashed lines. (**b**) is the corresponding comment’s arrival density plot of (**a**). It shows that the originally uneven and clustered distribution of arrival can be segmented into sections with similar comment arrival density. (**c**) Results of the process of recursive Fisher’s Exact Test for detecting multiple change points in a time series. The hypergeometric probability is calculated using Eq. (5) for each sub-segment. The minimum hypergeometric probability value of each segment is taken as the *p-value*. The *p-value* is then compared to a threshold ${\text{p}}_{\text{th}}$ to determine if the time point t is small enough to be considered as a change point $\upsilon$. If yes, the sub-segment will be cut into 2 sub-segments at the change point $\upsilon$. Then the same process will be repeated until the *p-value* of all sub-segments are larger than the threshold. The data label CP:i refers to the change point detected in the *i*th loop.

Here we show an example of the time series segmentation result (see Fig. 3). The details of segmentation method are provided in “Methods”—3. Segmentation of time series: multiple change point detection. After segmentation, the original time series for each emotion is segmented into different number of sections: *Anger*—651 sections, *Depression*—558 sections, *Fatigue*—208 sections, *Vigor*—659 sections, *Tension*—464 sections, *Confusion*—390 sections. The average section length is 14.3 h. The minimum section length is 0.5 h. The maximum section length is 55 days, due to very few comments related to *Fatigue* were posted from August to October 2020.

(2) Classify a time series section to detect endogenous and exogenous burst

As a pre-process we exclude “inactive periods” from the time series after the segmentation procedure. Inactive periods typically appear in the midnight (2 a.m.–5 a.m.), or after May 2020 when the COVID-19 crisis was under control in China (see Fig. 3b) so there are less COVID-19 related comments. The inactive periods are characterized by a very low submission rate of comments, and therefore no statistical property can be discussed. If a time series section partly or fully falls within 2 a.m.–5 a.m. (the condition can be set as start time ${t}_{\text{start}} \le \text{5}$ a.m. and end time ${t}_{\text{end}} \ge \text{2}$ a.m.), or if its average rate of comment arrival is lower than 1 comment per minute, we categorize the time series section as “inactive” and exclude it from our analysis.

Next, we define the categories which we will classify a time series segment into. When users post comments randomly and independently at a constant rate, the number of comments posted within a fixed time interval follows a Poisson process and the interevent time follows an exponential distribution. We define such scenario as scenario 1—random. When the posting behavior is neither random nor independent, the arrival of comments is clustered. This can result from an endogenous influence (such as preceding comments) or an exogenous influence (such as external news or events). When users are exposed to an endogenous influence, if the number of comments surge drastically, we define it as scenario 2—endogenous burst, elsewise as scenario 3—endogenous non-burst. Similarly, when users are exposed to an exogenous influence, if the number of comments surge drastically we define it as scenario 4—exogenous burst, elsewise as scenario 5—exogenous non-burst. In the following we will introduce the concept of classifying a time series into these five scenarios.

Conceptually, under different scenarios the distribution of interevent time can be different. Let ${t}_{j}$ be the time point at which *j*th comment is posted. Therefore, $\Delta {t}_{j}$= ${t}_{j}-{t}_{j-1}$ represents the posting time interval between *j*th and (*j *− 1)th comment.

When comments arrive randomly and independently (scenario 1—random), the number of comments within a fixed interval, ${r}_{t}$, follows a Poisson distribution with a constant average rate ${ \lambda }_{0}$, ${\text{r}}_{\text{t}} \sim \text{ Poi}({\lambda}_{0})$. The interevent time $\Delta {t}_{j}$ follows an exponential distribution $\Delta{\text{t}}_{\text{j}} \sim \text{ Exp}({\lambda}_{0})$. This can be tested using the Chi-square Test.

When the comment arrival is influenced by endogenous factors (scenario 2 and 3), the posting time series can be modeled as a self-modulation process where the probability of an event’s occurrence is dependent on precedent events. Such process is similar to Hawkes process or self-exciting (regulating) process^20,35,36^. Inspired by Takayasu and Takayasu^22^, we presume that the interevent time $\Delta {t}_{j}$ between *j*th and (*j *− 1)th comment depends on the average interevent time of precedent comments $\Delta {t}_{j-1}, \Delta {t}_{j-2}, \dots ,\Delta {t}_{j-k}$ posted over past *φ* period, where *φ* is a memory kernel and *k* is the number of comments in the past *φ* period. Namely, we assume that $\Delta {t}_{j}$ can be modeled using the following Eq. (1), in which ${b}_{j}$ is an independently and identically distributed (i.i.d.) random variable which follows the exponential distribution ~ $\text{Exp}(1)$. In this paper, the angle brackets <  > means the averaged value, namely, $<{\Delta t}_{j-1},{\Delta t}_{j-2}, \dots , {\Delta t}_{j-k}>=\frac{1}{k} \sum_{i=1}^{k}\Delta {t}_{j-i}$.

1 $$\Delta {t}_{j}={b}_{j}<{\Delta t}_{j-1},{\Delta t}_{j-2}, \dots , {\Delta t}_{j-k}>,$$

where

2 $${b}_{j} \sim \text{ Exp(}{1}\text{)}.$$

**Figure 4:**
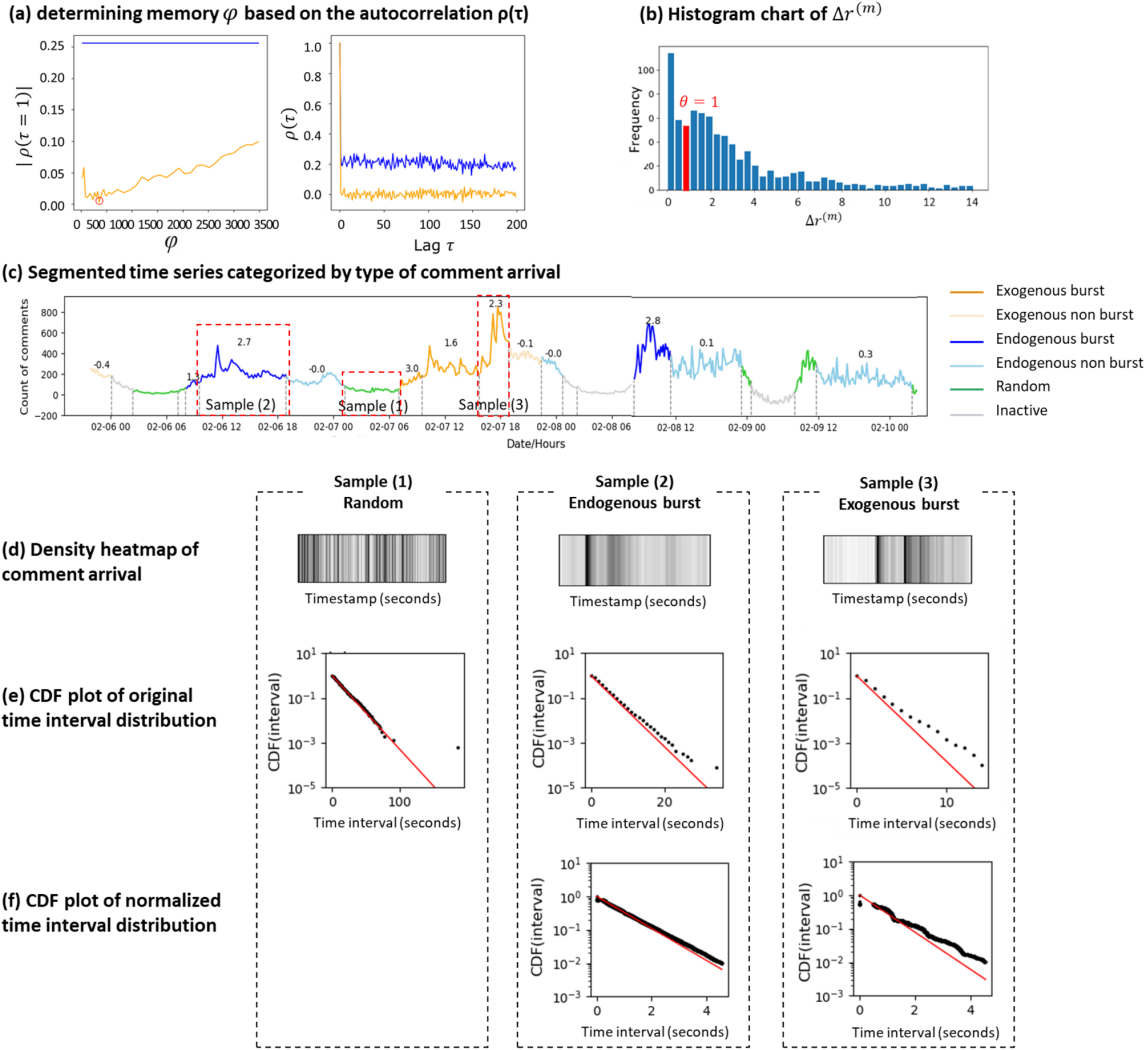
Example of the rule-based algorithm to classify time series segments. (**a**) An example of determining memory φ based on autocorrelation function ρ(τ). The blue line represents the autocorrelation value of the original time interval {$\Delta {t}_{j}$} and the orange line represents that of the normalized time interval {${b}_{j}$}. The first sub-figure shows result of |ρ(1)| based on different φ. |ρ(1)| reaches its minimum value close to 0 at φ = 360 s. This indicates that at φ = 360 s, the normalized time series is not autocorrelated anymore. The second sub-figure shows the autocorrelation function before and after normalization based on φ = 360 s. It obviously shows that the autocorrelation is removed after the normalization. (**b**) Histogram chart of $\Delta {{r}^{\left(m\right)}}$, the increment rate of number of comments. The bar in red (where $\Delta {{r}^{\left(m\right)}}$ = 1) represents the threshold value *θ*. (**c**) The result of burst analysis on a segmented time series (the unit time is 600 s), from which we picked 3 samples with different scenarios as circled in red box. The numbers marked above the line chart are increment rate $\Delta {{r}^{\left(m\right)}}$ of each segment. (**d**) Density heat map of comments arrival. X-axis represents the timestamp of comment arrival. The shorter the interval between the two comments, the darker the vertical lines are. (**e**) Semi-log CDF plot of the original time interval and a fitting exponential distribution line. X-axis represents the time interval in seconds and y-axis represents the cumulative distribution function of interval. The p-values of Chi-square test on samples 1, 2 and 3 are 0.23, $\text{6.58} \,\times \, {10}^{-212}$, and $\text{1.09} \times {10}^{-209}$, respectively. Because sample 1’s p-value is significant (> 0.05) enough to be accepted as an exponential distribution, it is classified as scenario 1-random. (**f**) Semi-log CDF plot of the normalized time interval and fitting exponential distribution line. The p-value of Chi-square test on sample 2 and 3 are $\text{1.43} \times {10}^{-3}$ and $\text{4.62} \times {10}^{-5}$, respectively. Because sample 2’s p-values is significant (> 0.0005) enough to be accepted as an exponential distribution and its increment rate is larger than *θ* = 1, sample 2 is classified as an endogenous burst while sample 3 is classified as an exogenous burst.

The value of memory period, *φ*, and the corresponding number of precedent comments, *k*, can be determined as follows. When there exists such dependency in a time series {$\Delta {t}_{j}$}, the autocorrelation function $\rho \left(\tau \right),$ especially $\rho \left(\tau =1\right)\ne 0$. Therefore, we can adjust the value of *φ* and *k* to calculate ${b}_{j}=\frac{\Delta {t}_{j}}{<\Delta {t}_{j-k}>}$ based on Eq. (1) and the corresponding autocorrelation function $\rho \left(1\right)$ based on Eq. (8). The optimal value of *φ* and *k* are the ones that remove (minimize) the autocorrelation value $\rho \left(1\right)$ of the time series {${b}_{j}$}. Details are described in the “Methods” section. Figure 4a shows an example of determining the value of *φ* based on the autocorrelation function.

If the normalized interevent time {${b}_{j}$} follows an exponential distribution (to be tested by the Chi-square test), it means that the original interevent time series {$\Delta {t}_{j}\}$ can be modeled by Eq. (1). Therefore, it can be classified to Scenario 2 and 3—endogenous. Otherwise, if {$\Delta {t}_{j}\}$ does not belong to Scenario 1, 2 or 3, we presume that the distribution of interevent time $\Delta {t}_{j}$ is influenced by exogenous factors such as external news or events.

However, not all time series classified to the endogenous or exogenous category are bursts (drastic increase). Some time series can be fluctuating or decreasing. Therefore, we need to calculate the increment rate of a time series. Only when the increment rate is larger than the threshold $\theta$, the time series segment can be classified as a burst. To find the threshold value *θ*, we calculate the increment rate (see Eq. (9)) for all endogenous and exogenous time series and plot the histogram chart (Fig. 4b). We take the first local minima of the histogram chart, where the increment rate equals to 1 as the threshold value (see the red bar in Fig. 4b). Figure 4c shows an example of the classification result where the increment value of each segment is annotated in the figure. We can see that the exogenous and endogenous segments with obvious increment are classified into burst, while those fluctuating or decreasing are classified into non-burst.

The five scenarios we introduce for categorization of segments are summarized as follows:

- Scenario 1: Random
  Interevent time follows exponential distribution $\Delta {t}_{j} \sim \text{Exp(}{\lambda}_{0}\text{)}$
- Scenario 2 and 3: Endogenous
  Interevent time $\Delta {t}_{j}$ is dependent on the averaged preceding interevent times and Eqs. (1) and (2) are fulfilled, which will be further classified into below two scenarios:
  - Scenario 2: Endogenous burst
  - Scenario 3: Endogenous non-burst
- Scenario 4 and 5: Exogenous
  The distribution of interevent time $\Delta {t}_{j}$ neither falls under scenario 1 nor scenario 2 and 3. It will be further classified into below two scenarios.
  - Scenario 4: Exogenous burst
  - Scenario 5: Exogenous non-burst

Based on the concept described above, we propose a non-parametric approach—the rule-based algorithm to categorize segments of time series based on the distribution of time intervals between consecutive comments as follows. The detailed method and algorithm are provided in the “Methods” section—4. Non-parametric rule-based algorithm to classify the time series sections. Figure 4c–f shows that our proposed non-parametric approach can segment a time series into homogenous sections and determine the type of arrival pattern.

### Burst features of different emotions and periods based on the COVID-19 related comments

**Figure 5:**
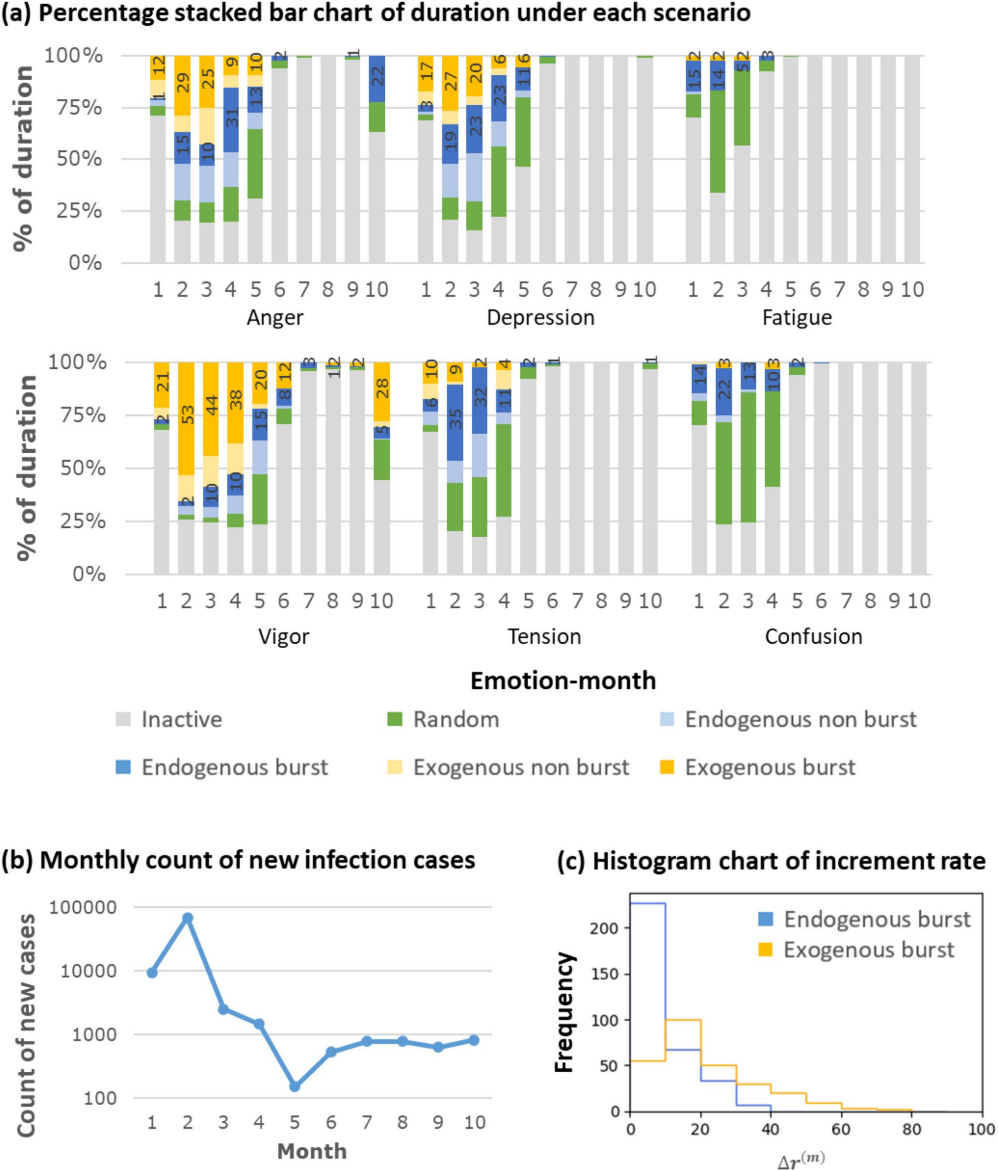
Comparison of comment’s arrival pattern across emotions and months. (**a**) Percentage bar charts summarizing the duration of each scenario for different months (x-axis) and emotions. The percentage of endogenous and exogenous period are marked in the plot. (**b**) Line chart showing the monthly count of new COVID-19 infection cases in China during January to October 2020. It can be observed that during January to April, which is the outbreak period in China, each emotion shows a longer burst period compared to other months. (**c**) Histogram chart of increment rate of the endogenous and exogenous burst. We can observe that the increment rate of the endogenous burst is lower than the exogenous burst. The average increment rates of endogenous and exogenous burst are 9.3 and 22.0, respectively.

Having segmented the 6 emotion time series and classified each time series section into the five scenarios, we summarize the duration of each scenario by month and emotion (see Fig. 5). We can observe that for each emotion, the burst period was longer from January to April compared to May to October. This trend corresponded to the count of COVID-19 new infection cases which peaked in February, underwent recovery from February to April and was under control since May. Therefore, collective emotion was considered to be affected by the COVID-19 situation.

Among all the emotions, *Vigor*, *Anger*, *Depression* had the longest burst duration which on average reached close to 50% during February to March in 2020. This indicates that these three emotions can easily form collective emotions during outbreak and recovery period of the COVID-19 in China.

For *Anger* and *Vigor*, the percentage of exogenous bursts was much higher than endogenous bursts, suggesting that *Anger* and *Vigor* were more likely triggered by external factors such as news and propaganda published by official accounts such as the governments or news presses. In contrast, *Depression* had a more balanced exogenous and endogenous bursts.

*Tension* had shorter burst periods compared to the above three emotions, but it still had more than 35% of time under burst status during February and March. Tension was more likely to be triggered by endogenous influence.

*Fatigue* and *Confusion* both had a comparatively low percentage of burst duration suggesting that they were less likely to form collective emotions. Duration of endogenous bursts was longer than that of exogenous bursts, which indicates that they were more influenced by other users’ comments instead of external news.

We are interested in the dynamics of emotion in terms of how the popularity of an emotion evolve during the endogenous and exogenous burst, and how different are the dynamics between different emotions. In the following we will further analyze the features of endogenous and exogenous bursts separately.

An endogenous burst is formed when users’ emotion and posting behavior are influenced by other users’ comments. We can use the self-modulation process model (Eq. 1) to explain the dynamics behind the emotion cascade. Based on the model, when the time interval between comments is short (or long), the arrival of the next comment also tends to be fast (or slow). Although the endogenous burst also represents an increase in the number of comments, the change is usually more gradual than the exogenous burst. We can observe from Fig. 5c that the increment rate of an endogenous burst is usually lower than an exogenous burst. Such self-modulation process can be normalized to a random process based on the memory period *φ* (Eq. 2), which suggests that the users are influenced of comments over precedent *φ* period.

**Figure 6:**
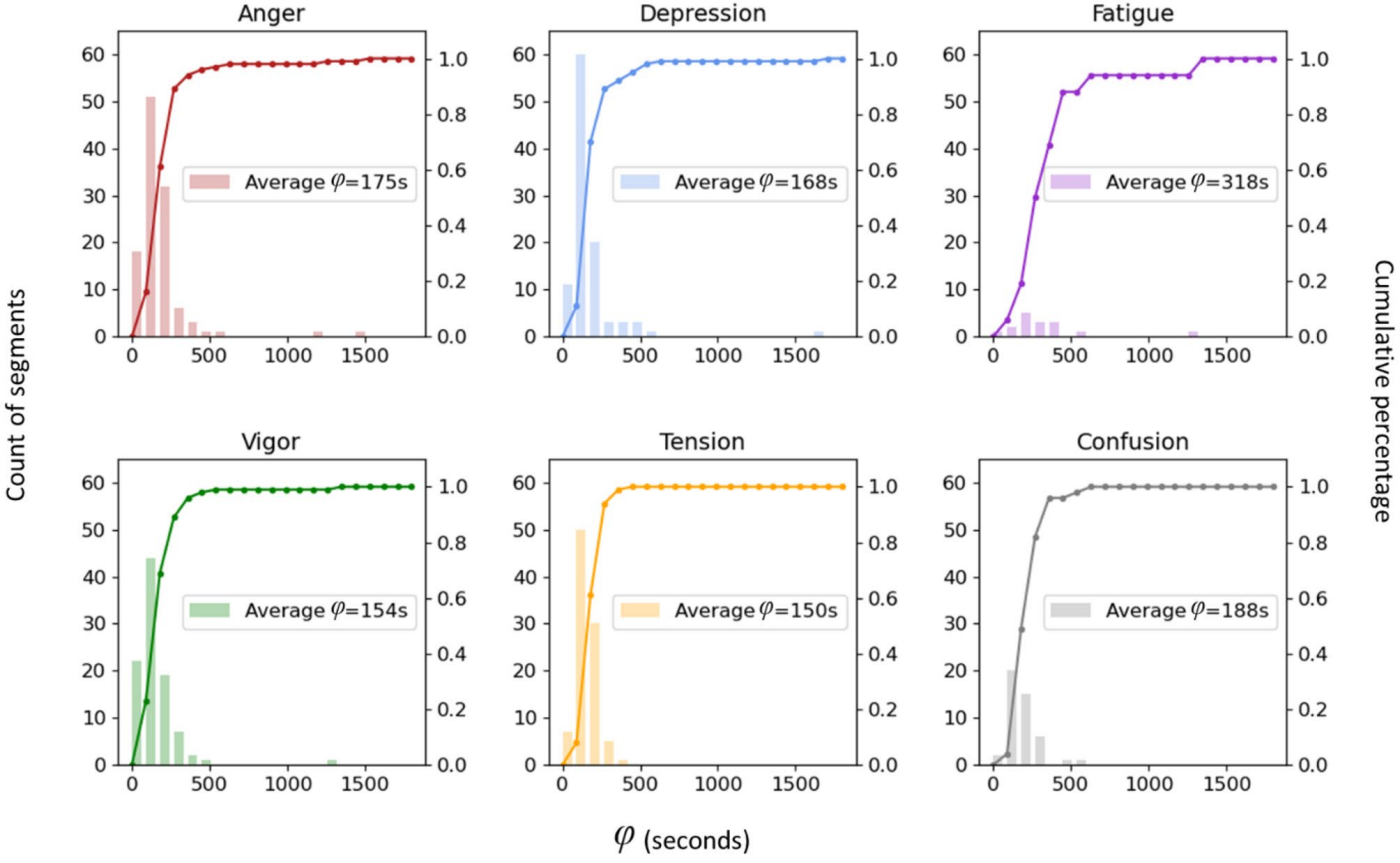
Histogram chart of memory period *φ* by emotion. The values of *φ* (ranges from 0 to 1800 s) are divided into 20 bins of the same size. The x-axis represents the memory period *φ* (in s) and y-axis represents the frequency distribution of the memory period. The dotted curves show the cumulative distribution function of the memory period. It is observed that the average memory periods for most emotions except for *Fatigue* are similar around 3 min.

In this model, the memory period *φ* is the key parameter to be determined empirically from the data. We plot a frequency distribution histogram in Fig. 6*.* We can observe that the memory period *φ* for most emotions are similar except for *Fatigue*. For most emotions, the average memory period ranges from 150 to 188 s, which is around 3 min. However, the average memory period of *Fatigue* is 318 s which is slightly higher. Therefore, we can conclude that during COVID-19 crisis, the popularity of a collective emotion triggered by peer influence is related to the short-term memory.

Next, we investigate the details of exogenous bursts. An exogenous burst can be understood as a sudden increase in comment arrival rates when influenced by external news or events, whose increment rate is comparatively higher than the endogenous burst (see Fig. 5c). The burst then decays gradually and returns to the non-active level as the popularity of the news fade away. In this paper, we apply a power-law function to model the dynamics of an exogenous burst. Let ${r}_{t}$ be the number of comments posted at time point *t* after an external news arrives at time point ${t}_{E}$. Let $\beta$ be the power-law decay exponent,

3 $${r}_{t}\propto {\left(t-{t}_{E}\right)}^{-\beta }.$$

**Figure 7:**
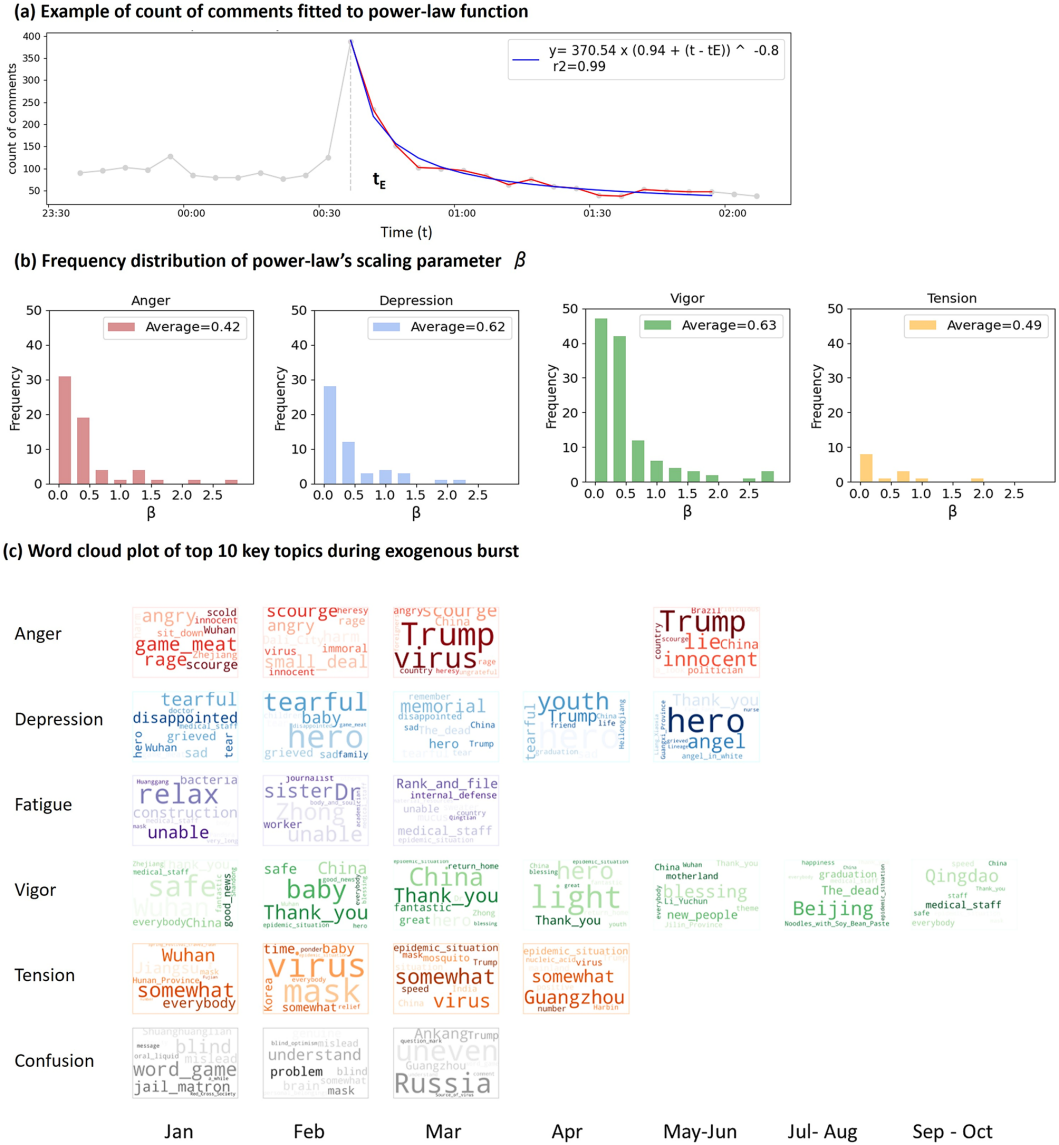
Features of exogenous burst. (**a**) An example of comment count time series that we categorized under exogenous burst. The post-burst part can be fitted nicely to power-law function with exponent equals to 0.8. (**b**) Histogram chart of power-law exponent *β* for each emotion (*Fatigue* and *Confusion* are not included here because they rarely have exogenous burst periods). Typical *β* value ranges from 0.42 to 0.63 for different emotions. (**c**) Word cloud chart visualizes the top 10 topic words of comments under exogenous period each month. The larger the font is, the more frequently the word occurs. Because the occurrence of exogenous bursts after May became rare, we visualized the key topics every two months after May. Those months with little or no exogenous burst period are left blank due to lack of data.

Here the power-law exponent $\beta$ is the key parameter to be determined based on empirical data analysis. We apply the nonlinear least squares to fit the number of comments ${r}_{t}$ to time elapsed after the external news being posted $\left(t-{t}_{E}\right)$. The unit time is set as 300 s. We find that 95% of the exogenous period can be fitted nicely by a power-law function. The average R-squared values for measuring the goodness-of-fit are as follows: *Anger*—0.77, *Depression*—0.80, *Tension*—0.81, *Vigor*—0.85 (the results of *Fatigue* and *Confusion* are excluded because they rarely have exogenous burst). Figure 7a shows an example of fitting data with a power-law function. Then we plot Fig. 7b to show the frequency distribution of power law exponent *β.* We can observe that the value of *β* for different emotions are as follows: *Anger*—0.42, *Depression*—0.62, *Vigor*—0.63, *Tension*—0.49.

The power-law decay exponent represents the persistence of collective response to an external influence^20^. Our fitting result reveals interesting difference in emotion persistence when users are exposed to exogenous news. *Anger* and *Tension* have a smaller power-law exponent which suggest that these two emotions are more persistent when exposed to external news related to COVID-19. Comparatively, *Depression* and *Vigor* has a larger power-law exponent which indicates that they are less persistent and fades away faster during the exogenous burst.

There are many other researches using a power-law function to model the relaxation of bursts after an external influence, for example, Sano et al*.*^19^ found that the burst of number of blog posts decayed after the 2011 Japan Tsunami with a power-law exponent of 0.67; Crane and Sornette^20^ found that the number of views on featured YouTube videos decayed with a power-law exponent of 0.6; Johansen and Sornette^37^ found that the popularity of papers relaxed obeying a power-law exponent of 0.58 after being introduced in an interview. However, none of these papers studied the difference of power-law exponent between different collective emotions. Therefore, our result shows some new findings related to the exogenous burst.

For exogenous bursts that are triggered by external news or events, we also investigate what news topics attracted users’ attention. We gather comments that form the exogenous bursts and use Term Frequency Inverse Document Frequency (TF-IDF)^38^ method to extract one topic word from each comment. Then we visualize 10 most frequently occurred topics (with the highest TF-IDF value) for each emotion and each month as shown in Fig. 7c.

## Methods

### Dataset

Weibo is a leading social platform in China which has been widely used during the COVID-19 outbreak period for information sharing and communication. The active Weibo user reached 241 million during the first quarter 2020, increasing by 15% compared to the same period in 2019. Like Twitter, it is a public platform where users can post and comment in short text. In this paper we use two datasets:

1. The main dataset in which all comments are related to COVID-19. It contains 27.2 million comments that were collected during 1st January to 31st October 2020.
2. The control dataset that is not related to COVID-19. It contains 9.2 million comments that were collected during 1st September to 31st December 2019, before the COVID-19 outbreak.

**Figure 8:**
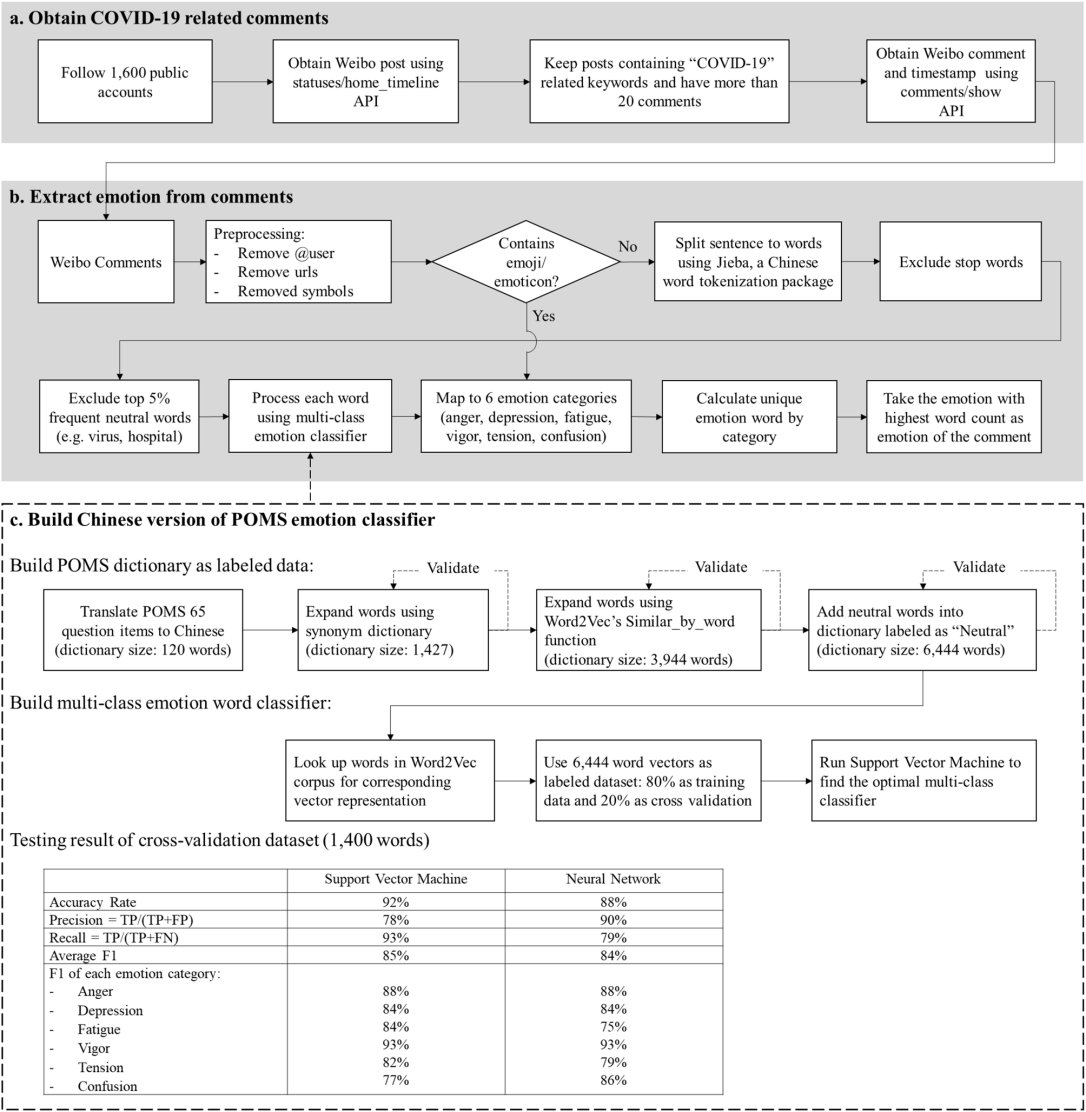
Process to detect emotion from the COVID-19 related comments. (**a**) Process to obtain the COVID-19 related comments using open Weibo API. The COVID-19 related keywords are {疫情 (epidemic/pandemic), 肺炎 (pneumonia), 感染 (infection), 病毒 (virus), 新冠 (coronavirus), 隔离 (quarantine), 核酸 (PCR test), COVID}. (**b**) The algorithm to pre-process comment data, tokenize into words and then label each comment with either a POMS emotion or neutral tag by looking at each word’s emotion classification. (**c**) The process to build the Chinese version of POMS emotion classifier combining dictionary-based and machine learning algorithm. The training result shows that the performance between Support Vector Machine and Neural Network are similar in general. We adopted Support Vector Machine which gives better accuracy rate.

Both datasets were collected using Weibo open API (https://open.weibo.com/wiki/API). More specifically, we first collected the COVID-19 related posts published by 1600 public accounts (news organizations, government, influential individuals, etc.) and then obtained comments under these posts (see Fig. 8). Note that because we want to study collective emotions rather than individual emotions, only posts with more than 20 comments are included. Detailed data collection process and profile of 1600 verified public accounts are provided in the Supplement 1.

### Extract POMS emotions: emotion word classification

There are various approaches to categorize words, sentences, or paragraphs according to a pre-defined emotion category based on the classification of emotions. The most common methods are dictionary-based approaches that look up emotion words in a pre-defined dictionary^31,32,39–42^, rule-based approaches that define a rule of how to use available information (e.g. linguistic features, emoticons, etc.) for predicting the emotion categories^43,44^, and machine learning algorithm that uses corpus to build classification model^4,18,45,46^. In this paper, we use words and emotion categories defined in POMS questionnaire. However, the pure dictionary-based approach contains a limited range of words and therefore is not suitable for analyzing social media data which are written in informal languages such as slangs, abbreviations, or new words.

Our research adopts dictionary-based approach but expands it using machine learning algorithms (Fig. 8). We first created a Chinese version of POMS dictionary (the corpus has been provided in Supplement 2). Then we used this dictionary as labeled data for training a multi-class emotion classifier which can automatically classify a word to either neutral category or one of POMS emotion categories. To run the machine learning training, we looked up each word in a Word2Vec corpus (developed by Tencent AI lab that provides 200-dimension vector representation for 8 million Chinese words^47^) to get a corresponding word vector. The Word2Vec is a technique that maps a word to a high dimensional vector space based on its semantic similarity to other words^48^. This feature has been used by researchers to classify emotions presuming that words with similar emotion are closer in the vector space^45,46,49^.

Our training objective is to find the optimal boundaries between seven clusters: six POMS emotion clusters—*Tension*, *Anger*, *Vigor*, *Fatigue*, *Depression*, *Confusion*, and neutral cluster. We ran both Support Vector Machine (using Scikit-learn package^50^) and Neural Network algorithm based on 6444 labeled words, 80% of which were used for training and 20% of which were used for cross validation. Figure 8a shows that both algorithms performed well, but the Support Vector Machine algorithm yielded a slightly higher accuracy rate. Therefore, we used a model trained by Support Vector Machine to classify new words.

We processed two datasets using the algorithm described in Fig. 8b and extracted emotions from comments. More details of this emotion classification method are explained in Supplement.

### Segmentation of time series: multiple change point detection

The change point detection algorithms have been widely studied and applied in fields like signal processing^51^, financial market^21,52^ and climatology^53^. They are categorized into either parametric or non-parametric approaches. The parametric approach fits the time series data into one or a selection of known distribution functions, then find a segmentation point where the distribution changes. The non-parametric approach compares the homogeneity of the time series before and after a time point and tests if it is significant enough to be treated as a change point.

In this paper, we focus on the non-parametric algorithm to reduce computational complexity. There are various non-parametric algorithms developed for detecting multiple change points. Wilcoxon Rank Statistic^54^ compares the homogeneity of two samples’ population mean ranks and extends it to detect multiple change points using dynamic programming. Maximum Likelihood Estimation^55^ which treats time series as binary data uses Bayesian information criterion to find number of change points and dynamic programming to get locations of change points. Wild Binary Segmentation^56^ localizes multiple change point problem by recursively running single change point detection in subsegments based on CUSUM (cumulative sum) statistics. Density Ratio Estimation^57^ adopts non-parametric Gaussian kernel model to calculate the density ratio of sample data before and after each time point, then choose those points as change points if the density ratios exceed a pre-defined threshold.

We adopted a non-parametric method based on Fisher's Exact Test proposed by Sato and Takayasu^21^. The advantage of Fisher’s Exact Test is that the p-value is directly calculated based on statistics and therefore is more accurate for any data size.

We localize the multiple change points detection by starting with a single change point detection. Let ${r}_{t}$ be the count of comments belonging to the same emotion at time point *t*, where *t* ∈ [0, *T*]. Let *F*(${r}_{t}$:[*t*_1_, *t*_2_]) be the probability distribution function of comment count in the interval [*t*_1_, *t*_2_]. We use Fisher’s Exact Test to check homogeneity of time series before and after time point $\upsilon$. The hypothesis test is defined as follows:

- H0 (time point $\upsilon$ is not a change point): $F\left({r}_{t}: \left[0,\upsilon \right] \right)= F\left({r}_{t}: \left[\upsilon +1, T\right]\right)$
- H1 (time point $\upsilon$ is a change point): $F\left({r}_{t}: \left[0,\upsilon \right]\right)\ne F\left({r}_{t}: \left[\upsilon +1, T\right]\right)$

To apply this statistical test, we count the number of points *a, b, c* and *d* shown in the Table 1 contingency table, by comparing each data point ${r}_{t}$ to *h* and comparing its time point *t* to $\upsilon$. *h* is a constant with value $\mathrm{min}({r}_{t})\le h \le max({r}_{t})$. Its value can be determined by trying different *h* and adopting the one that generates minimum p-value based on Fisher’s Exact Test. To reduce computation complexity, we divide the range [$\mathrm{min}\left({r}_{t}\right),max({r}_{t})$] evenly into ten deciles and take each decile as potential *h* value.

**Table 1:** Contingency table of Fisher’s Exact Test.

Count of time point *t*	$0\le t \le \upsilon$	$\upsilon < t \le T$	Row subtotal
${r}_{t}>h$	*a*	*b*	*R*_1_ = *a* + *b*
${r}_{t}\le h$	*C*	*d*	*R*_2_ = *c* + *d*
Column subtotal	*C*_1_ = *a* + *c*	*C*_2_ = *b* + *d*	*a* + *b* + *c* + *d*

Given the contingency table denoted as ${X}_{\upsilon }$, we calculate the hypergeometric probability at time point $\upsilon$.

4 $${\text{P}}\left( {{\text{X}}_{\upsilon } } \right) = \frac{{\left( {\begin{array}{*{20}c} {\text{a}} \\ {{\text{a + d}}} \\ \end{array} } \right) \times \left( {\begin{array}{*{20}c} {\text{b}} \\ {{\text{b + d}}} \\ \end{array} } \right)}}{{\left( {\begin{array}{*{20}c} {{\text{a + b}}} \\ {{\text{a + b + c + d}}} \\ \end{array} } \right)}}.$$

Then we sum up hypergeometric probabilities p(*Y*) that are smaller than p(${\text{X}}_{\upsilon }$) based on contingency tables of all possible combinations of *a*, *b*, *c*, *d* that returns the same subtotals.

$${\text{For}}\,Y = \left\{ {Y\;{\text{is}}\;{\text{a}}\;2 \times 2\;{\text{contingency}}\;{\text{table}},\sum\limits_{{j = 1}}^{2} {Y_{{ij}} } = R_{i} ,\sum\limits_{{i = 1}}^{2} {Y_{{ij}} } = C_{i} ,R_{i} \in X_{\upsilon } ,C_{j} \in X_{\upsilon } } \right\}$$,

5 $${\text{P}}\left( {{\text{X}}_{\upsilon } } \right) = \sum\limits_{{{\text{k}} = {\text{0}}}}^{{{\text{min}}\left( {{\text{a,d}}} \right)}} {\frac{{\left( {\begin{array}{*{20}c} {{\text{a}} - {\text{k}}} \\ {{\text{a}} + {\text{d}}} \\ \end{array} } \right) \times \left( {\begin{array}{*{20}c} {{\text{b}} + {\text{k}}} \\ {{\text{b}} + {\text{d}}} \\ \end{array} } \right)}}{{\left( {\begin{array}{*{20}c} {{\text{a}} + {\text{b}}} \\ {{\text{a}} + {\text{b}} + {\text{c}} + {\text{d}}} \\ \end{array} } \right)}}} + \sum\limits_{{{\text{k}} = {\text{1}}}}^{{{\text{min}}\left( {{\text{b,c}}} \right)}} {\frac{{\left( {\begin{array}{*{20}c} {{\text{b}} - {\text{k}}} \\ {{\text{b}} + {\text{d}}} \\ \end{array} } \right) \times \left( {\begin{array}{*{20}c} {{\text{a}} + {\text{k}}} \\ {{\text{a}} + {\text{c}}} \\ \end{array} } \right)}}{{\left( {\begin{array}{*{20}c} {{\text{a}} + {\text{b}}} \\ {{\text{a}} + {\text{b}} + {\text{c}} + {\text{d}}} \\ \end{array} } \right)}}} ,$$

6 $${\text{p - value}} = \mathop {{\text{min}}}\limits_{{\upsilon ,{\text{h}}}} {\text{P}}({\text{X}}_{\upsilon } ),\;{\text{where}}0 \le \upsilon \le {\text{T}}\;{\text{and}}\;{\text{ min}}({\text{r}}_{{\text{t}}} \le )\;{\text{h }} \le {\text{max}}({\text{r}}_{{\text{t}}} )$$

If the p-value is smaller than the significance level ${p}_{th}$, we reject the null hypothesis and treat the time point $\upsilon$ as a change point for time series {${r}_{t}$}. Otherwise, we accept $\upsilon$ as the change point. The significance level ${p}_{th}$ is determined by shuffling the time series {${r}_{t}$} randomly *N* times and taking the minimum p-value generated from *N* trials as ${p}_{th}$ (in this paper we shuffled the time series for *N* = 1000 times). This indicates that for a random time series (no change points), the probability Pr $({\text{p - value}} \le {\text{p}}_{{{\text{th}}}} ) = \frac{1}{{\text{N}}} \to 0\,{\text{for}}\,{\text{large}}\,{\text{N}}$. We repeat the same procedures recursively on time series segments to the left and right of the detected change point until all segments’ p-value are higher than ${p}_{th}$.

Empirically, it is still computational costly to run the hypothesis test on 10 months’ time series even after aggregating per 600 s. To reduce the data size, we process 3 days’ time series at once and then combine the last section with the following 3-day time series to maintain continuity. As a result, data size can be confined within around 500 time points per calculation. We also observe that the significance level ${p}_{th}$ tends to be larger when time series {${r}_{t}$} has wider range. We shuffle time series 1000 times and got ${p}_{th}={10}^{-4}$ when max $\left\{{r}_{t}\right\}>50$ and set ${\text{p}}_{\text{th}} = {10}^{-6}$ when max $\left\{{r}_{t}\right\}\le 50$.

### Non-parametric rule-based algorithm to classify the time series sections

The rule-based algorithm consists of 4 steps.

Step (1): Check if the original interevent time {$\Delta {t}_{j}$} belongs to the random category (if it is exponentially distributed).

We start with testing if the time series in a segment is randomly and independently distributed using Chi-square test. Let $F\left(\Delta {t}_{j}\right)$ be the cumulative distribution function (CDF) of real-valued interevent time $\Delta {t}_{j}$ in the segment, and $\widehat{F}\left(\Delta {t}_{j}\right)=\mathrm{exp}\left(- {\lambda}_{0}\Delta {t}_{j}\right)$ be the CDF of exponential distribution that is fitted to $F\left(\Delta {t}_{j}\right)$ using non-linear least squares.

The Chi-square test is defined as follows:

- H_0_ ($\Delta {t}_{j}$ follows an exponential distribution):$F\left(\Delta {t}_{j}\right)=\widehat{F}\left(\Delta {t}_{j}\right)$
- H_1_ ($\Delta {t}_{j}$ does not follow an exponential distribution): $F\left(\Delta {t}_{j}\right)\ne \widehat{F}\left(\Delta {t}_{j}\right).$

The test statistics ${\chi }^{2}$ can be calculated using Eq. (7).

7 $$\chi ^{2} = \sum\limits_{{j = 1}}^{n} {\frac{{\left( {F\left( {\Delta t_{j} } \right) - \hat{F}\left( {\Delta t_{j} } \right)} \right)^{2} }}{{\hat{F}\left( {\Delta t_{j} } \right)}}} ,$$

where *n* represents the total number of $\Delta {t}_{j}$ data points in the time series segment. Based on ${\chi }^{2}$ and degree of freedom (*n *− 1), the p-value can be calculated using the Chi-squared distribution. The p-value is then compared to the significance level 0.05 to determine if reject or accept the null hypothesis. If the ${\text{p - value}}\,{\text{is}}\,{\text{larger}}\,{\text{than}}\,0.05$, then the null hypothesis H_0_ is accepted, we conclude that $\Delta {t}_{j}$ follows an exponential distribution and the arrival of comment is random not showing burst features.

Step (2): If {$\Delta {t}_{j}$} is not randomly distributed, remove the autocorrelation from {$\Delta {t}_{j}$} and build the normalized time series {${b}_{j}$}.

This step aims to find the optimal value of $\varphi$, the correlation period, and *k*, the number of precedent comments posted within period $\varphi$. The autocorrelation function can be calculated as follows.

8 $$\rho \left( \tau \right) = \frac{{\sum\nolimits_{{j = k + \tau + 1}}^{n} ( b_{j} \; - < b_{j} > )(b_{{j - \tau }} \; - < b_{{j - \tau }} > )}}{{\sum\nolimits_{{j = k + 1}}^{n} {\left( {b_{j} \; - < b_{j} > } \right)^{2} } }},$$

where ${b}_{j}=\frac{\Delta {t}_{j}}{<\Delta {t}_{j-k}>}$ (Eq. 1), $\Delta {t}_{j}$ is the time interval between (*j *− 1)th and *j*th comment, ${\text{j}} \in [{\text{1}},{\text{n}}]$. $\tau$ is the time lag (here we set the unit lag time *τ* as 600 s). *k* is the number of comments arrived right before *j*th comment over the past $\varphi$ period.

We gradually increase $\varphi$ from 0 to up to 3600 s (assuming user’s memory is shorter than 1 h), calculate corresponding normalized time interval {${b}_{j}$} and its autocorrelation function $\rho \left(\tau \right)$ until we find a local minimal $\rho \left(1\right)$ which is within distance 0.01 from the origin.

Step (3): Check if {${b}_{j}$} follows an exponential distribution using the Chi-square test (similar to step (1)).

Here we set Chi-square test’s significance level at 0.0005 instead of 0.05 because in Eq. (1) for calculating the average time interval of comments posted within a period *φ*, we assume that the memory period *φ* is fixed for the time series segment to reduce computation complexity. As described in the 2nd step, the value of *φ* may be varied so we lower the significance level to mitigate the impact of this assumption on the result. If the Chi-square test’s null hypothesis is accepted, meaning the normalized time intervals follow the exponential distribution, then we conclude that the comment arrival is caused by an endogenous self-modulation effect (scenario 2 or 3). Otherwise, the segment is considered as exogenous (scenario 4 or 5).

Step (4): Calculate the increment rate of a time series $\Delta {{r}^{\left(m\right)}}$ and categorize it to a burst or a non-burst

Let ${r}_{s}^{\left(m\right)}$ be the number of comments of the *s*^e^ interval in the *m*th segment, where $s\in \left[\mathrm{0,1},2,\dots ,{s}_{m}\right]$. The unit of s is fixed as 600 s. We define the increment rate of number of comments in the *m*th segment, $\Delta {{r}^{\left(m\right)}}$, by comparing the largest number of comments in the *m*th segment to the average value of (*m *− 1)th segment, < ${r}_{s}^{\left(m-1\right)}$>, or to the number of comments of the *m*th segment’s starting point, ${r}_{0}^{\left(m\right)}$, whichever is larger.

9 $$\Delta {{r}^{\left(m\right)}}=\frac{\mathrm{max}\left(\left\{{r}_{s}^{\left(m\right)}\right\}\right)-\mathrm{max}\left(<{r}_{s}^{\left(m-1\right)}>,{r}_{0}^{\left(m\right)}\right)}{\mathrm{max}\left(<{r}_{s}^{\left(m-1\right)}>,{r}_{0}^{\left(m\right)}\right)}.$$

The rule-based algorithm to classify a time series section into the five scenarios can be summarized using the following pseudocode:
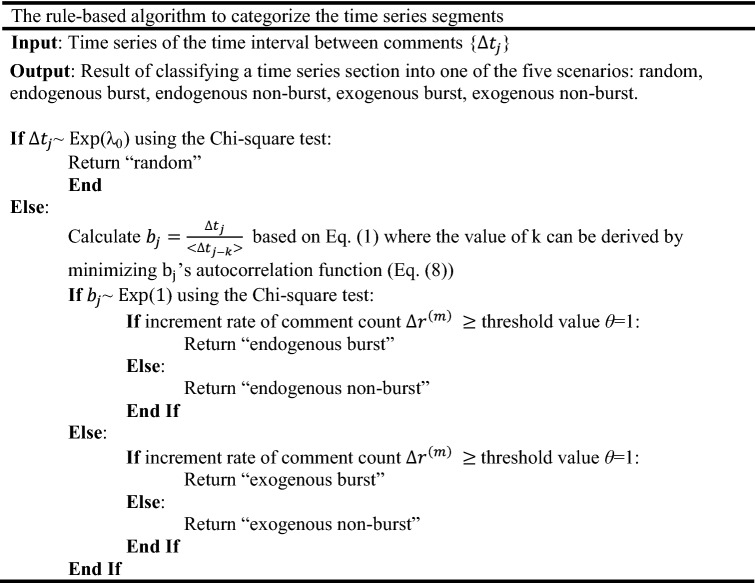


## Conclusion and discussion

In this paper, we have proposed a novel approach to detect endogenous and exogenous emotion bursts from 27.2 million Weibo comments. We use a machine-learning algorithm to extract multi-class emotions from comment texts, segment the emotion time series into sections and then use a rule-based algorithm to identify the bursts. Our approach is non-parametric and therefore suitable for analyzing dataset of any size.

The analysis result reveals interesting differences in the burst feature between collective emotions. *Vigor*, *Anger* and *Depression* had significantly longer burst duration than *Fatigue* and *Confusion* especially during the COVID-19 outbreak period. *Vigor* and *Anger* bursts were more triggered by exogenous influence, while *Tension*, *Fatigue* and *Confusion* bursts were more triggered by endogenous influence. For the endogenous burst, we show that the word-of-mouth dynamics can be modeled by a self-modulation process during which emotions cascade based on a short-term memory period *φ*. The values of *φ* are similar for most emotions at around 3 min while *Fatigue* has a longer memory period of 5 min. For exogenous bursts, we show that the drastic surge of number of comments followed by relaxation can be modeled by a power-law function whose decay exponent *β* represents the persistence of the external influence. For emotions bursts triggered by the exogenous factors, we find that the values of *β* are smaller for *Anger* (0.42) and *Tension* (0.49), and larger for *Depression* (0.62) and *Vigor* (0.63), suggesting that the external influence on *Anger* and *Tension* are more persistent.

To our knowledge, the burst analysis based on multi-class collective emotions during COVID-19 is a novel research topic. We have shared the detailed empirical data analysis method to make it easily reproducible by other researchers. It can be an interesting future study to compare the burst features between different countries (such as the length of endogenous and exogenous bursts, the burst modeling parameters and key topic words), which may show differences in collective emotion response based on different COVID-19 situations, cultural backgrounds and prevention measures.

Our proposed burst detection method is not only applicable for analyzing social media data but can also be applied to analyze financial markets where each trader’s behavior may be influenced by other traders (endogeneity) or external news (exogeneity). It may also be applied to analyze burst phenomena in complex systems such as computer networks and servers. For example, the internet traffic burst that threats network security and affects user experience may be caused by endogenous factors (such as uneven and clustered usage) or external factors (cyber-attack or other extraordinary events). Our method can help to detect abnormality, identify the root causes of the bursts, and improve system performance.

The limitations of this paper are as follows. Firstly, due to the limited API usage and access provided by Weibo, we only obtained comments under posts published by 1600 official accounts. Compared to the large user population in Weibo, our results may not represent the whole user group. By using the comment data, we may constrain the topics being discussed among the users by the contents published by the official accounts. Ideally, our method could be better applied to the full posts data for detecting emotion bursts in the real public sphere. Secondly, we assumed that under the endogenous burst scenario, the rate of arrival is dependent on the average rate of comments over past $\varphi$ period where $\varphi$ is a constant value for the given time series segment. This assumption is based on the intuition that users tend to read the latest comments over a certain period and then post a comment. However, the actual user behavior and the way that the comments are presented to users (for example, the most liked or interacted comments are promoted to the top) may be more dynamic and more complicated. Thirdly, we analyzed the six emotions independently, but not looking into the synergy between emotions. We are interested in exploring this topic as a future work.

## Supplementary Information

Supplementary Information 1.

Supplementary Information 2.

## Data Availability

The datasets analyzed during the current study are not publicly available due to Weibo open API policy (keeping personal data confidential), but aggregated and anonymized data are available from the corresponding author on reasonable request. Similar data can be obtained using Weibo API (https://open.weibo.com/wiki/API). Details are provided in in the Supplementary 1 (3. Data Collection Process).
