# Supplementary material for: Classification of endogenous and exogenous bursts in collective emotions based on Weibo comments during COVID-19
Source: Sci Rep. 2022 Feb 24;12:3120. doi: 10.1038/s41598-022-07067-w (PMC8873493; doi:10.1038/s41598-022-07067-w)
Supplement: Supplementary file 1 — Supplementary Information 1. [file 41598_2022_7067_MOESM1_ESM.pdf]

SI Appendix 1:

Classification of endogenous and exogenous bursts in collective emotions based on Weibo comments during COVID-19

Qianyun Wu, Yukie Sano, Hideki Takayasu, Misako Takayasu

1. Building Chinese version of POMS dictionary.

POMS (Profile of Mood States) is a questionnaire based psychological scaling tool for measuring emotion status. It contains 6 emotion categories: *Tension*, *Anger*, *Vigor*, *Fatigue*, *Depression*, and *Confusion*. Traditionally, a participant is given a questionnaire consisting of 65 questions, each formed by an emotion word belonging to one of the emotion categories. The participant is asked to rate a score from 0 to 5 depending on how strong they can feel the emotion. In our research we make use of the POMS questionnaire as guideline to classify emotion words.

To our best knowledge, POMS questionnaire has not yet been officially translated into Chinese. Therefore, to make use of the questionnaire we first translated emotion words given in the questionnaire to Chinese using Cambridge dictionary (one English word can be translated to different Chinese words with similar meanings). In total we got 120 Chinese words which serve as stem words.

Then we expanded the stem words using the synonyms database provided by Social Computing and Information Research Center, China Harbin Institute of Technology (https://www.ltp-cloud.com/intro_en). For example, given the stem word "hate", all its synonyms under the same group Gb10A01 will be included into our dictionary as shown in **Fig.S1**. This expansion results in on total 1,427 words which we think is still not enough to be used as the labeled training dataset.

Gb10A01= 讨厌 厌恶 嫌恶 憎恶 痛恶 恶 厌 憎 嫌 烦 厌烦 腻烦 腻味 腻 头痛 作呕 倒胃口 深恶痛绝 疾首蹙额 掩鼻而过
Hate, disgust, dislike, bored, headache....
Gb10B02= 仇视 仇恨 憎恨 憎恶 疾 交恶 反目成仇 狭路相逢 反目为仇 嫉恨 忌恨 结仇 会厌 夙嫌 亲痛仇快
Hate, hatred, turn into enmity, start feud, jealous...

Supplementary Figure S1. Example of synonyms database which groups synonyms words under the same group code. For example, all words under Gb10A01 are of similar meanings. We look a word up in the database and extract words under the same group as synonyms.

Therefore, we continue to expand the dictionary using Word2Vec's Similar_by_word function. Word2Vec is a natural language processing technique that map a word to a high dimensional vector space based on proximity to its context words. Therefore, synonyms that often show in similar context will tend to be positioned closer to each other. In this research we use Word2Vec corpus provided by Tencent AI Laboratory (https://ai.tencent.com/ailab/nlp/en/embedding.html), which has been trained using large-scale texts from news, webpages and books. The Similar_by_word function can find the 10 closest

words in the vector space which are likely to be the stem word's synonyms. This function expanded the dictionary size to 3,900 words.

During each of above steps, we manually check each word to verify the classification accuracy.

Other than the 6 POMS emotion categories, we also created a "*Neutral*" category for classifying unemotional words. We extracted the top frequent words that have been used in the comments and manually picked 2,500 words to build the "neutral" category.

Therefore, in total our training dataset- the POMS emotion dictionary – contains 3,944 emotion words and 2,500 neutral words. The numbers of emotion words per category are summarized in **Supplement Table S1**.

Supplement Table S1. Number of Emotion Words by Category

Emotion	*Anger*	*Depression*	*Fatigue*	*Vigor*	*Tension*	*Confusion*	*Neutral*
Number of words in dictionary	779	814	393	818	683	457	2500

2. Multi-class emotion word classifier

Before starting the training, we map each word in the POMS emotion dictionary to a 200-dimension vector using the Word2Vec corpus provided by Tencent AI Laboratory[2]. We add 1 to the beginning of the word vector and define it as $X_w = [1, x_1, x_2, \dots, x_{200}]$. In total there are 6,444 words in the emotion dictionary. We randomly select 80% of the words to build the training dataset and 20% to build the cross-validation dataset.

In this paper, we used two machine learning algorithms – Neural Network and Support Vector Machine (SVM) to train the multi-class emotion classifier. Then we adopt the algorithm that gives better results after testing on cross-validation data.

The Neural Network algorithm is a machine learning algorithm widely used in training non-linear classifiers. Conceptually, it consists of *N* layers, with first layer as the input layer and the last layer as output layer while the rest are hidden layers. The number of layers *N* and the number of elements $n^{(L)}$ in each layer *L* are adjustable to fit the modeling need. In this research, we adopt N=4, $n^{(2)}$=25, $n^{(3)}$=25 after trying different options. **Supplement Fig.S2** shows the structure of the neural network. The model can be written as follows.

Let g(z) be the sigmoid function:

$$g(z) = \frac{1}{1 + e^{-z}} \tag{1}$$

Let $\theta^{(l)}$ be the weight matrix between layer (l-1) and l. Let $a_k^{(l)}$ be the kth parameter in layer l.

Given the input vector Xw, let $\theta^{(1)} = \begin{bmatrix} \theta_{1,0}^{(1)} & \cdots & \theta_{1,200}^{(1)} \\ \vdots & \ddots & \vdots \\ \theta_{25,0}^{(1)} & \cdots & \theta_{25,200}^{(1)} \end{bmatrix}$ be the weight matrix of first layer. Each parameter $a_k^{(2)}$ in the second layer can be calculated as:

$$a_k^{(2)} = g\ (z_k^{(2)}) \tag{2}$$

where $z_k^{(2)}$ can be calculated by the dot product of matrix $\theta^{(1)}$ and vector Xw:

$$\begin{bmatrix} z_1^{(2)} \\ z_2^{(2)} \\ \vdots \\ z_{25}^{(2)} \end{bmatrix} = \theta^{(1)}\ Xw^T \tag{3}$$

$$= \begin{bmatrix} \theta_{1,0}^{(1)} & \cdots & \theta_{1,200}^{(1)} \\ \vdots & \ddots & \vdots \\ \theta_{25,0}^{(1)} & \cdots & \theta_{25,200}^{(1)} \end{bmatrix} \begin{bmatrix} 1 \\ x_1 \\ \vdots \\ x_{200} \end{bmatrix} \tag{4}$$

$$= \begin{bmatrix} \theta_{1,0}^{(1)} + \theta_{1,1}^{(1)} x_1 + \theta_{1,2}^{(1)} x_2 + \cdots + \theta_{1,200}^{(1)} x_{200} \\ \theta_{2,0}^{(1)} + \theta_{2,1}^{(1)} x_1 + \theta_{2,2}^{(1)} x_2 + \cdots + \theta_{2,200}^{(1)} x_{200} \\ \vdots \\ \theta_{25,0}^{(1)} + \theta_{25,1}^{(1)} x_1 + \theta_{25,2}^{(1)} x_2 + \cdots + \theta_{25,200}^{(1)} x_{200} \end{bmatrix} \tag{5}$$

Having obtained all the parameters $a_k^{(2)}$ in the second layer, we use the same method as described in Eq.(1)-(5) to calculate the parameters in third layer $a_k^{(2)}$ and output layer h.

$$h = \theta^{(3)}\ a_k^{(3)} \tag{6}$$

The output layer h is a 7-dimensional vector, where each element represents the probability of a word belonging to one of the emotions *e*: *h* = [P(*Anger*), P(*Depression*), P(*Fatigue*), P(*Vigor*), P(*Tension*), P(*Confusion*), P(*Neutral*)]^T. The emotion with highest probability will be taken as the emotion $\widehat{y_w}$ predicted by the model.

Our training objective is to find the optimal weight matrix $\theta^{(l)}$ that can minimize the average cost function $J(\theta)$ for all *m* words in the training set. For each word, the cost function can be calculated by comparing the output *h* vector with vector *y* converted from actual emotion that the word belongs to. Let vector *y* be the actual emotion classification of a word. For example, for a given word with *Anger* emotion, the corresponding vector *y* should be [1, 0, 0, 0, 0, 0, 0]^T. The cost function of this word can be calculated as follows.

$$j_w(\theta) = Cost(h, y) = \sum_{e=1}^{7} -y_e \times \log(h_e) - (1 - y_e) \times \log\ (1 - h_e) \tag{7}$$

$$J(\theta) = \frac{1}{m} \sum_{w=1}^{m} j_w(\theta) \tag{8}$$

We use Gradient Descent algorithm to run the optimization for 30,000 iterations and calculate the optimal parameter $\theta^{(l)}$ that minimizes $J(\theta)$.

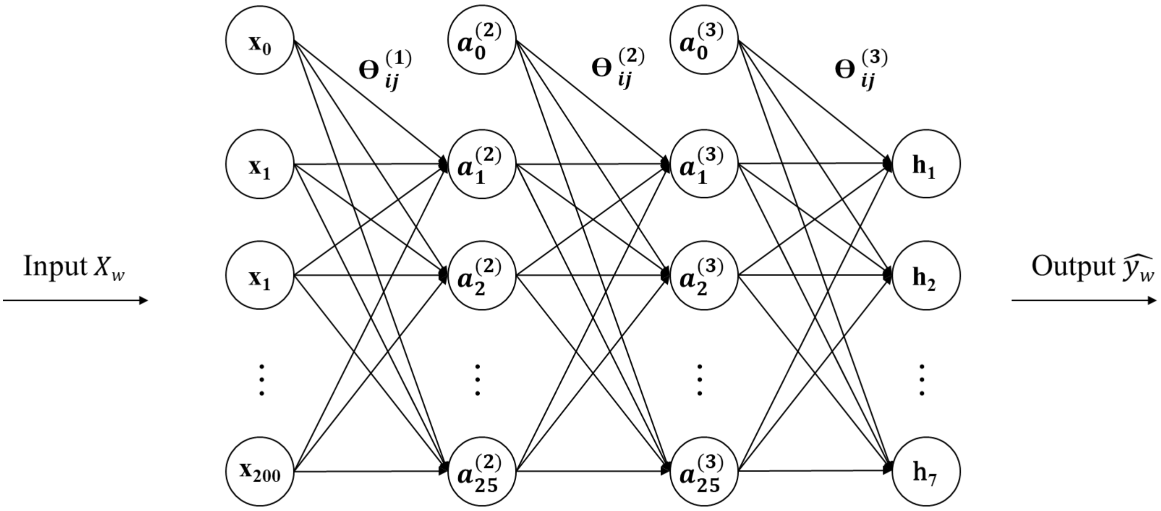

Supplementary Figure S2. Neural Network Model Illustration

The other machine learning algorithm we used was SVM. It is often combined with Word2Vec for training multi-class classifier. In this research we use the Python package Scikit-learn (Scikit-learn: Machine Learning in Python, Pedregosa et al., JMLR 12, pp. 2825-2830, 2011) to run the analysis.

Let us briefly introduce the algorithm. Similar to Neural Network algorithm, SVM also built the mathematical prediction model with input word vector and parameters θ_{SVM}, then it looks for optimal θ_{SVM} that can minimize the cost function. To apply it to the multi-class classification, we run SVM separately for predicting each emotion and then choose the emotion with highest prediction value.

The mathematical model can be described as follows. Let θ_{SVM} be the parameter vector and x_w be the word vector. y_w represents if the predicted emotion is equivalent to actual emotion *e* of word w. For example, if the model is predicting *Anger* emotion while the actual emotion is *Depression*, y_w=0. The SVM's cost function $J_e(\theta)$ can be written as

$$J_e(\theta) = C \times \sum_{w=1}^{m} [y_w \times cost_1({\theta_{SVM}}^T x_w) + (1 - y_w) \times cost_2(\theta_{SVM} x_w)] + \frac{1}{2}\sum_{w=1}^{m} \theta_j^2 \qquad (9)$$

where C is a constant and θ_j represents a large margin to keep the distance between training data and decision boundary balanced.

$cost_1$ and $cost_2$ are approximations of logistics function.

$$cost_1 = \begin{cases} 0, & if\ \theta_{SVM}{}^T x_w \geq 1 \\ a_1 - a_1 x, & if\ \theta_{SVM}{}^T x_w < 1\ (a_1 > 0) \end{cases} \tag{10}$$

$$cost_2 = \begin{cases} 0, & if\ \theta_{SVM}{}^T x_w \leq -1 \\ a_2 + a_2 x, & if\ \theta_{SVM}{}^T x_w > -1\ (a_2 > 0) \end{cases} \tag{11}$$

Because we have only 200 features, which is relatively small compared to the training dataset size. Here we apply the Radial Basis Function Kernel (RBF kernel) to adjust the nonlinear decision boundary to improve the prediction result. With the RBF kernel, the cost function $J_e(\theta)$ can be modified as

$$J_e(\theta) = C \times \sum_{w=1}^{m} [y_w \times cost_1(\theta_{SVM}{}^T f_w) + (1 - y_w) \times cost_2(\theta_{SVM}{}^T f_w)] + \frac{1}{2} \sum_{w=1}^{m} \theta_j^2 \tag{12}$$

where f_w is the kernel function to increase the complexity of original input vector x_w. When using the Scikit-learn package, we chose RBF kernel, and set C=2, γ = 2 as the training parameter which can generate the best output after trying different values. Then we ran the package to look for the optimal θ based on input word vectors.

3. Data Collection Process

The data collection process can be illustrated in **Supplement Fig.S3**.

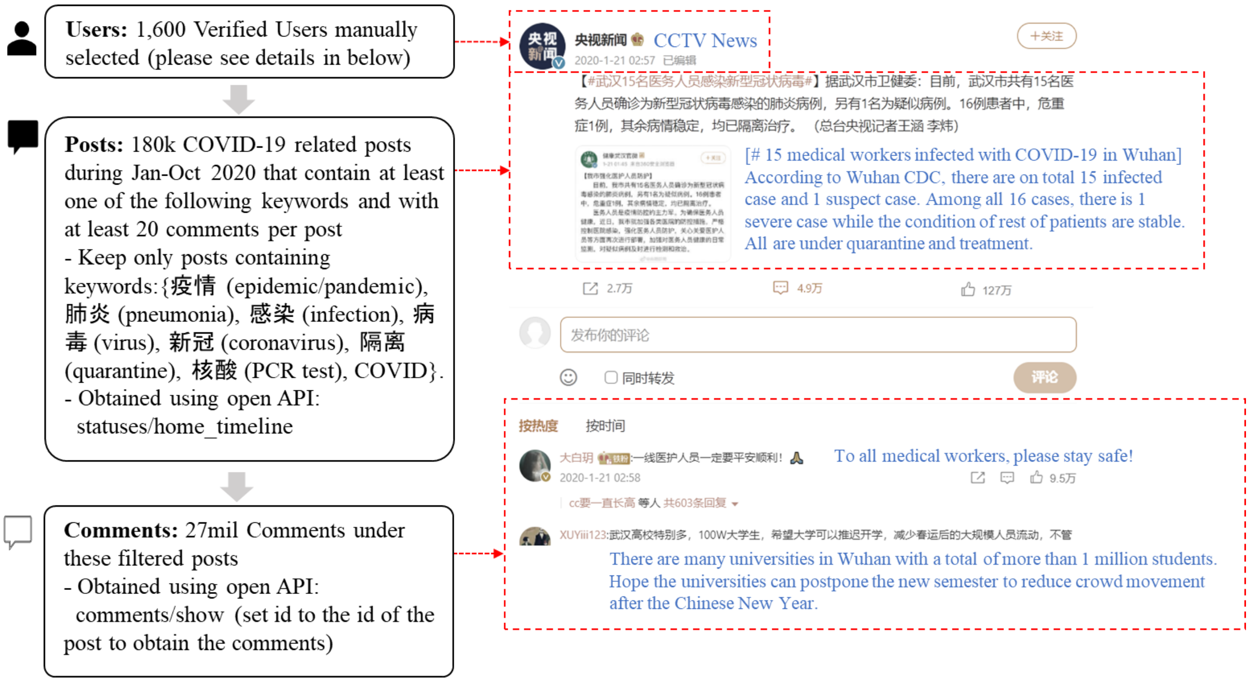

Supplementary Figure S3. Process to collect COVID-19 related comments.

The overall process is to manually select and follow 1,600 public accounts that consist of government, news organization, organizations, company, influential individuals accounts. Though there are many more other public accounts, we selected the ones that publish more COVID-19 related contents. We then collect the posts (similar to Tweets) by these accounts given that the post contains COVID-19 related keywords and has at least 20 comments (more likely involving discussions). Next, we obtain the comments under these posts, which we take as dataset for our research.

We used open API (https://open.weibo.com/wiki/API%E6%96%87%E6%A1%A3/en) provided by Weibo platform to obtain the posts and comments. Note that Weibo standard open API may only obtain data posted within 24 hours and has limited access rate per hour. Therefore, multiple developer accounts may be required to obtain sufficient post or comment data.

4. Profile of verified public accounts

To give more details on the profile of 1,600 verified public accounts, we summarized the distribution of number of followers per verified user shown in **Supplement Fig.S4**. It can be observed that all verified users have at least 1000 followers. More than 85% of the verified users have more than 100,000 followers. This indicates that they are the influencers in the Weibo platform and are more likely to trigger comments from other users.

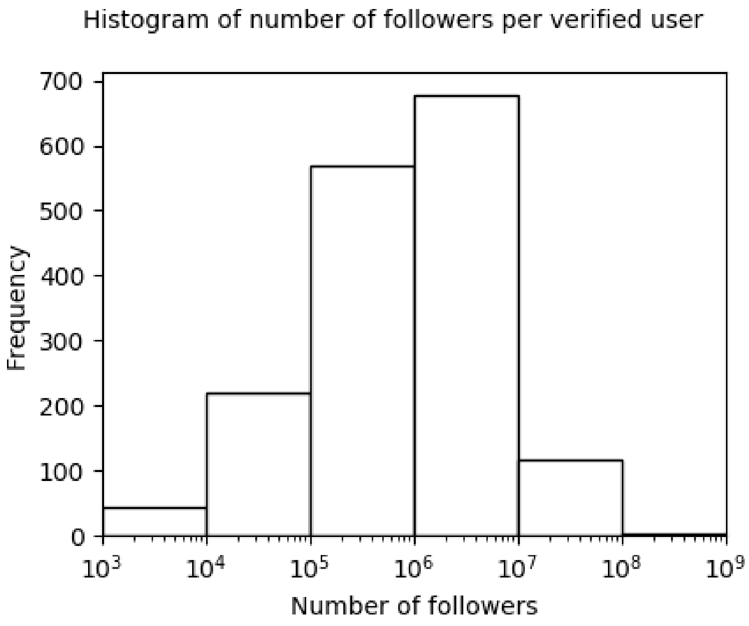

Supplementary Figure S4. Distribution of number of followers per verified users.

The verified accounts can be categorized into 4 main categories: influential individual, news organization, government, and others. We show the number of verified accounts by each category and give some examples for each category in **Supplement Table S2**.

Supplement Table S2. Number of verified accounts by account category.

**Account Category**	**Number of verified accounts**	**Examples**
Influential individual	1090	Luo Yonghao (entrepreneur), Jia Zhangke (film director), Yingguo Baojie (freelance writer)
News organization	285	China Daily, CCTV News, People's Daily
Government	177	China police, Communist Youth League, United Nations
Others (company, organization, school, website, etc.)	48	Red Cross Society of China, China mobile, Wuhan University
